# Differences in Competitive Ability between Plants from Nonnative and Native Populations of a Tropical Invader Relates to Adaptive Responses in Abiotic and Biotic Environments

**DOI:** 10.1371/journal.pone.0071767

**Published:** 2013-08-16

**Authors:** Zhi-Yong Liao, Ru Zhang, Gregor F. Barclay, Yu-Long Feng

**Affiliations:** 1 Key Laboratory of Tropical Forest Ecology, Xishuangbanna Tropical Botanical Garden, Chinese Academy of Sciences, Kunming, Yunnan Province, China; 2 University of Chinese Academy of Sciences, Beijing, China; 3 Department of Life Sciences, University of the West Indies, St. Augustine, Trinidad and Tobago; 4 College of Bioscience and Biotechnology, Shenyang Agricultural University, Shenyang, Liaoning Province, China; Wuhan Botanical Garden, Chinese Academy of Sciences, Wuhan, China, China

## Abstract

The evolution of competitive ability of invasive plant species is generally studied in the context of adaptive responses to novel biotic environments (enemy release) in introduced ranges. However, invasive plants may also respond to novel abiotic environments. Here we studied differences in competitive ability between *Chromolaena odorata* plants of populations from nonnative versus native ranges, considering biogeographical differences in both biotic and abiotic environments. An intraspecific competition experiment was conducted at two nutrient levels in a common garden. In both low and high nutrient treatments, *C. odorata* plants from nonnative ranges showed consistently lower root to shoot ratios than did plants from native ranges grown in both monoculture and competition. In the low nutrient treatment, *C. odorata* plants from nonnative ranges showed significantly lower competitive ability (competition-driven decreases in plant height and biomass were more), which was associated with their lower root to shoot ratios and higher total leaf phenolic content (defense trait). In the high nutrient treatment, *C. odorata* plants from nonnative ranges showed lower leaf toughness and cellulosic contents (defense traits) but similar competitive ability compared with plants from native ranges, which was also associated with their lower root to shoot ratios. Our results indicate that genetically based shifts in biomass allocation (responses to abiotic environments) also influence competitive abilities of invasive plants, and provide a first potential mechanism for the interaction between range and environment (environment-dependent difference between ranges).

## Introduction

Biological invasions are one of the major threats to natural ecosystems [1]. However, the reasons why invasive plant species grow more aggressively in their nonnative ranges than in their native ranges are still not well elucidated [2]–[4]. Many studies have found that invasive species escape from natural enemies of their native ranges when being introduced into new ranges [5], [6]. Such release from enemies may increase competitive ability of invasive plants simply by decreasing ecological restrictions, which in turn may cause evolutionary changes [7]–[9]. The evolution of increased competitive ability (EICA) hypothesis posits that invasive plants tend to decrease costly defense traits after long liberation from natural enemies, reallocating the newly available resources to growth and reproduction [2]. The EICA hypothesis predicts that invasive genotypes are less resistant to enemies and more competitive than native genotypes. Nevertheless, studies focused on both predictions of the EICA hypothesis have produced inconsistent results [10]. For example, similar or even greater defense is found in some invasive plants compared with their native conspecifics [11]–[14], although many studies find lower defense in plants from nonnative ranges compared with plants from native ranges [7], [9], [15], [16].

Most of the studies testing the EICA hypothesis were conducted in conditions without competition or with interspecific competition [10]. Growth without competition may not be an adequate measure of competitive ability [10], [17], [18]. In addition, local adaptation, species-specific interactions, and possible coevolution may play roles in alien plant invasions, and using only one or a few native competitors from either native or nonnative ranges may lead to biased conclusions in interspecific competition experiments [3], [10], [19]. In contrast, intraspecific competition experiments, which directly compare the difference in competitive ability between plants from nonnative and native ranges, can test the EICA hypothesis better [10], [17]. But few studies have been conducted to test the EICA hypothesis using intraspecific competition (but see [17], [20]).

It is well known that plant competitive dynamics and defense depends on resource availability [21]. Invasive *Acacia saligna* outperforms native *Protea repens* under high- and moderate-nutrient conditions but not under low nutrient conditions [22]. Invasive *Phragmites australis* outcompetes its native counterparts under nutrient-rich conditions but not under limited nutrient conditions [23]. Environment may affect relative competitive performance of species by influencing biomass allocation to different organs [24]. With increasing availability of soil nutrients, competition shifts from belowground to aboveground resources, favoring plants with high light capture ability [23]. However, few studies have compared the differences in competitive ability between plants from nonnative versus native ranges of introduced plants under varied environments (but see [20], [25], [26]), and to our knowledge no study has explained the differences in biomass allocation.

In addition, plant defenses may also be related to nutrient availability [27]. It is important to compare the differences in defense between plants from nonnative and native ranges of introduced plants under different nutrient conditions.

In this study, we explored the biogeographical differences in competitive and defensive abilities of *Chromolaena odorata* (L.) R. M. King & H. Robinson (Asteraceae), a perennial weed or subshrub by comparing plants of populations from its native versus nonnative ranges at two nutrient levels in a common garden with plants grown in pots. It is native to the Americas from southern USA to northern Argentina and is one of the worst invasive species in the humid (sub)tropics of the old world [28]. It tends to colonize and form dense monocultures in disturbed habitats including roadsides, waste places, forest trails, and abandoned fields [29], [30]. Soil nutrient availability is high under *C. odorata* plants [31], [32], and increased availability of soil nutrients significantly promotes growth of the invader [33]. More than 200 natural enemies have been documented for *C. odorata* in its native ranges, while only a few phytophagous insects feed on *C. odorata* in its introduced ranges [34], [35]. We hypothesized that *C. odorata* plants from nonnative ranges have higher competitive ability (competition-driven decreases in height and biomass were less) and invest fewer resources in defense traits than plants from native ranges. We focused on the potential effects of soil nutrient availability on above biogeographical differences and its underlying mechanisms. To determine plant defensive ability, we measured leaf toughness and the contents of cellulose, hemicellulose, and phenolics [36].

## Materials and Methods

### Ethics Statement

No specific permits were required for the described field studies. The location is not privately owned or protected in any way and the field studies did not involve endangered or protected species.

### Study Site

The experiment was carried out in Xishuangbanna Tropical Botanical Garden, Chinese Academy of Sciences (21°56′ N, 101°15′ E, 600 m above sea level), located in the southern part of Yunnan Province, southwest China. *Chromolaena odorata* occurs locally at the study site. Here the mean annual temperature is 21.7°C, with a mean of 25.3°C in the hottest month (July) and 15.6°C in the coolest month (January). The mean annual precipitation is 1,557 mm with a dry period from November to April.

### Seed Collection and Experimental Design

Seeds of *C. odorata* were collected in 2009 from eight populations in each range (Table S1 in File S1). Sample populations were more than 100 km apart from each other. The populations sampled in native and nonnative ranges were from similar latitudes to minimize the potential effect of latitude [37]. Within each population, seeds were collected from five plants that were at least 20 m apart from one another, and saved separately in paper envelopes (five seed families per population).

In June 2009, seeds of each seed family of each population were sown separately in seedling trays in a shade house with 50% irradiance. The germination medium was a mixture of river sand and forest topsoil (1∶1). In August 2009, when the seedlings were about 5 cm tall, similar-sized vigorous seedlings were transplanted into 15 L pots located under shade netting that allowed 50% irradiance. For monoculture, each pot contained one seedling, and 10 seedlings per population (two from each seed family) were planted. For competition, each pot contained two seedlings, one from each range and 10 cm apart from each other, and 80 seedlings per nonnative population (sixteen from each seed family) were competed with 10 seedlings from each of the eight native populations (two from each seed family). Populations from the same range were not competed with each other. Pots contained a mixture of 70% topsoil of a secondary forest (dominated by *Phoebe lanceolata* and *Castanopsis indica*; excluding plant litters and stones) and 30% river sand. Topsoil was used to provide a natural supply of macro- and micronutrients and the river sand provided a texture with adequate drainage and facilitated harvest of fine roots. After two weeks of growth in 50% irradiance, all seedlings were grown in full sunshine by removing the shade net. Seedlings were divided randomly into two groups; one was fertilized monthly from September to December with granules of compound fertilizer (nitrogen:phosphorous:potassium, 15∶15:15; Nitrophosha^R^, BASF, Belgium) at the rate of 4 g per pot, and the other group was the unamended low nutrient control. To eliminate the potential effects of position on growth, pots were assigned to five blocks in the garden, and each block contained 160 pots, 32 pots in monoculture (one pot per population per nutrient treatment) and 128 pots in competition (eight nonnative populations×eight native populations per nutrient treatment). All pots were placed on bricks to prevent roots from growing out and were turned every 20 days to change relative positions of seedlings to reduce the potential effect of position on growth. The seedlings were watered daily with a drip irrigation system. Pots were weeded when necessary.

### Measurements

Fully expanded leaves were collected in October 2009 from five plants per population (one per seed family) per nutrient treatment grown in monoculture for measuring defense traits. Leaf toughness was determined by measuring the maximal force required to puncture the leaf using a penetrometer with a 0.8 mm diameter flat-tipped needle (Chatillon 250 GF; Ametek, Largo, FL, USA). Each leaf was punctured three times and three leaves per sample plant were measured. Leaves were dried at 60°C to constant mass and powdered for measuring phenolics, cellulose, and hemicellulose. Total phenolics were measured using Folin Ciocalteu method [38]. Gallic acid was used as the standard. Hemicellulose was hydrolyzed using 2 mol/L hydrochloric acid, and the reduced sugar in the hydrolysate was determined using DNS (3,5-dinitrosalicylic acid) colorimetric analysis [39]. Cellulose was hydrolyzed using 60% sulfuric acid, and the glucose in the hydrolysate was measured using anthrone colorimetric analysis [40]. Glucose was used as the standard when measuring cellulosic contents.

In August 2009 (two weeks after transplantation), initial plant height was measured. In January 2010 (20 weeks after initial nutrient treatment), all plants were harvested after measuring final height. Samples were separated into roots and shoots. Roots were collected in sieves after washing off excess soils. Root and shoot materials were then oven-dried at 60°C to constant mass, and weighed. Total biomass and root to shoot ratio were calculated. Competitive ability was measured by the percent change in performance (height and total biomass) caused by competition, i.e. [(*P*_comp_−*P*_single_)/*P*_single_]×100%, where *P*_single_ is plant performance when grown without competition and *P*_comp_ is plant performance when grown with competition. In this study, *P*_single_ was the average of all replicates per population per treatment and *P*_comp_ was the value for each individual sample.

### Statistical Analyses

Effects of range, soil nutrient treatment, and their interaction on all variables including competitive ability were analyzed using two-way nested ANOVAs, with range, nutrient treatment, and their interaction as fixed factors, and population nested within range and interaction of population nested within range by nutrient treatment as random factors. The difference between ranges in each variable measured in each nutrient treatment was further tested using one-way nested ANOVAs, with range as the fixed factor, and population nested within range as a random factor. Initial plant height was used as a covariate when analyzing plant height, total biomass, and percent changes in these variables; total biomass was used as a covariate when analyzing the root to shoot ratio. Data were ln-transformed to meet the requirements of ANOVA (normal distribution and homogeneity of variances) when necessary. All analyses were done using SAS V8 (SAS Institute, Cary, North Carolina, USA).

## Results

### Biogeographical differences in Growth, Competitive Ability, and Biomass Allocation

**Figure 1:**
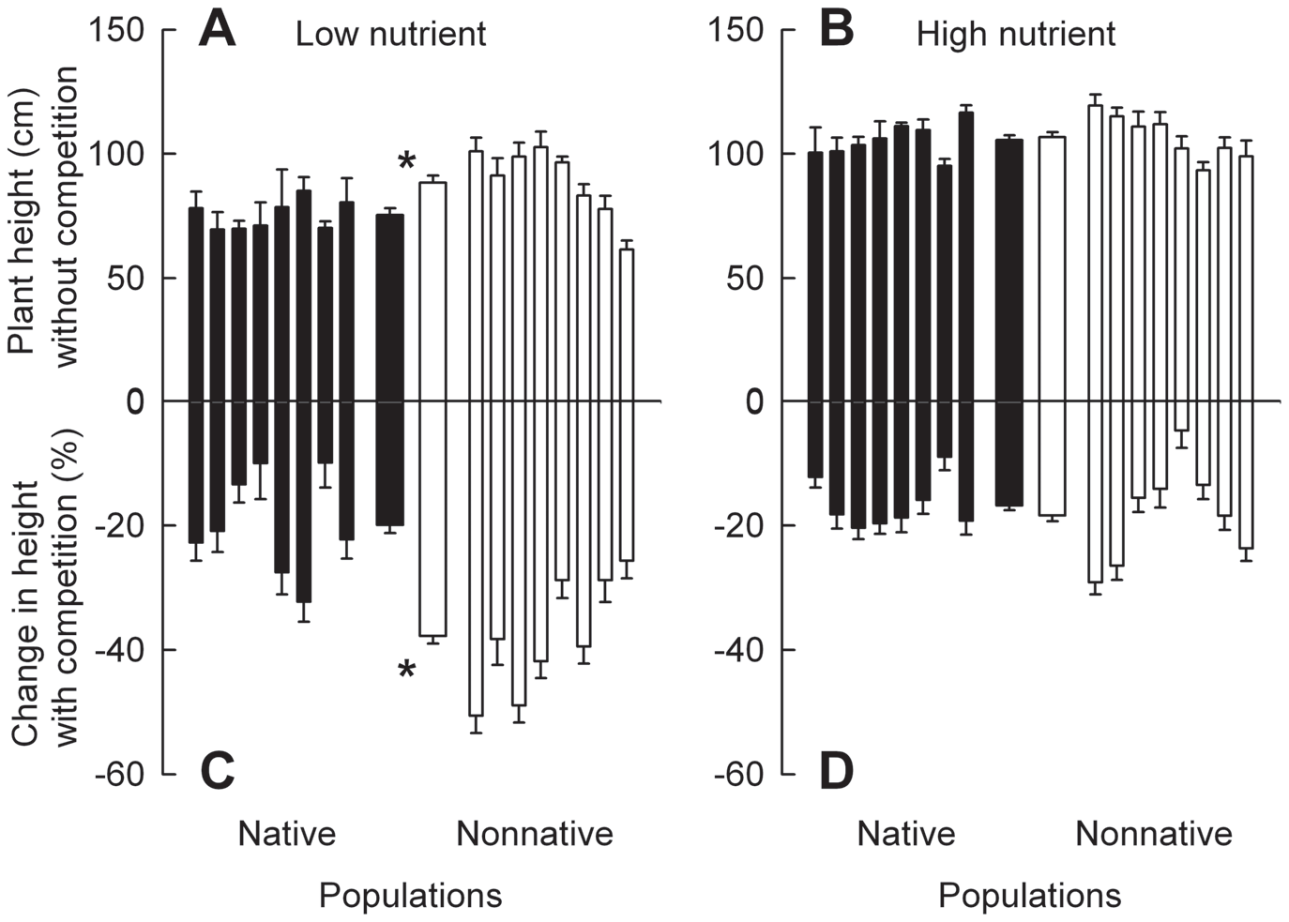
Differences in height between *Chromolaena odorata* plants of the populations from native versus nonnative ranges. Panels A and B were for plants grown in monoculture, and panels C and D were for plants grown in competition. Panels A and C were for plants grown at low nutrient level, and panels B and D were for plants grown at high nutrient level. Narrow bars depict means and SE for each population; two thicker bars in the center are means and SE for all populations from each range. * indicates significant difference between ranges at each nutrient level according to one-way nested ANCOVA (*P*<0.05; Tables S2, S4 in File S1).

**Figure 2:**
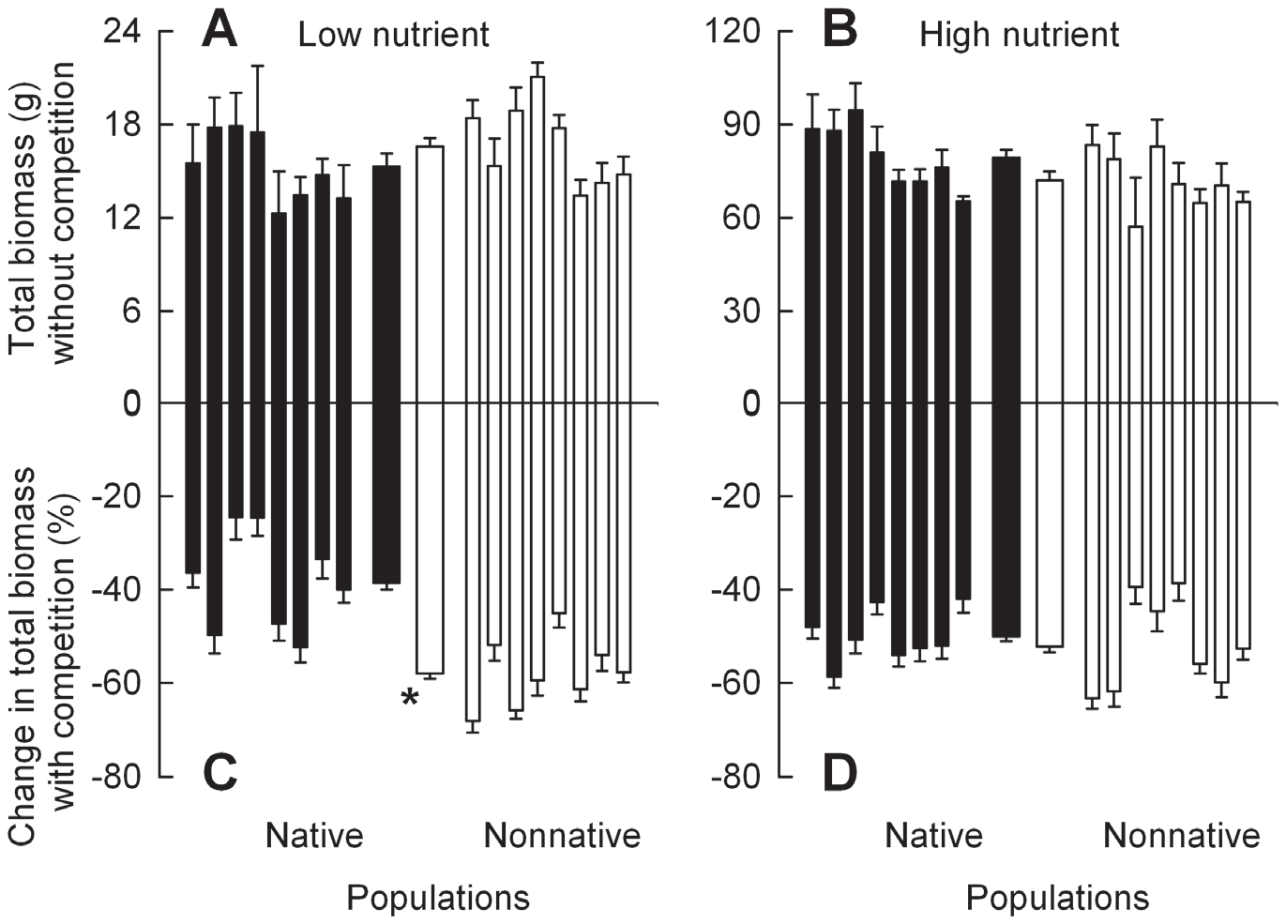
Differences in biomass between *Chromolaena odorata* plants of the populations from native versus nonnative ranges. Panels A and B were for plants grown in monoculture, and panels C and D were for plants grown in competition. Panels A and C were for plants grown at low nutrient level, and panels B and D were for plants grown at high nutrient level. Narrow bars depict means and SE for each population; two thicker bars in the center are means and SE for all populations from each range. * indicates significant difference between ranges at each nutrient level according to one-way nested ANCOVA (*P*<0.05; Tables S2, S4 in File S1).

When grown without competition, *C. odorata* plants of the populations from nonnative ranges were 17.5% taller than plants of the populations from native ranges in the low nutrient treatment, but total biomass did not differ significantly between plants from the two ranges (Figs. 1A; 2A; Table S4 in File S1). Nutrient addition significantly increased height and total biomass of *C. odorata* plants from both ranges (Table S2 in File S1). However, neither height nor total biomass differed significantly between *C. odorata* plants of the populations from nonnative versus native ranges in the high nutrient treatment (Figs. 1B; 2B). Competition significantly decreased height and total biomass of *C. odorata* plants from both ranges at both nutrient levels (Figs. 1C, D; 2C, D). Importantly, competition-driven decreases in these variables were significantly greater for plants of the populations from nonnative ranges than for plants of the populations from native ranges in the low nutrient treatment, but not in the high nutrient treatment. When grown with competition, *C. odorata* plants of the populations from native ranges were 9.6% taller and 33.8% heavier than plants of the populations from nonnative ranges in the low nutrient treatment (Fig S1 in File S1; Tables S6, S7 in File S1). In the high nutrient treatment, plants of the populations from native ranges were still higher in total biomass but not in height. The results are consistent with the noteworthy interactions between range and nutrient level (Table S2 in File S1).

**Figure 3:**
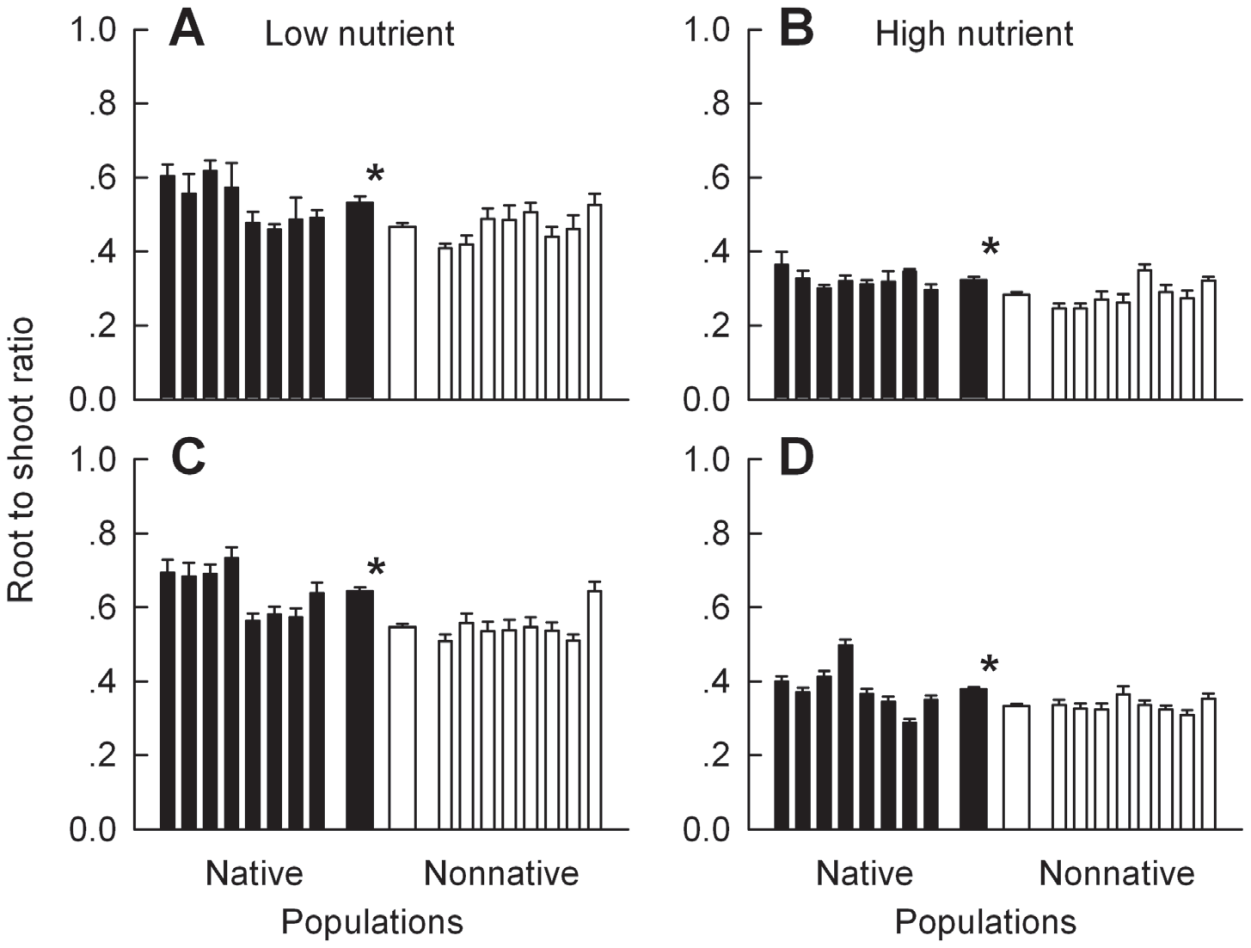
Differences in root to shoot ratios between *Chromolaena odorata* plants of the populations from native versus nonnative ranges. Panels A and B were for plants grown in monoculture, and panels C and D were for plants grown in competition. Panels A and C were for plants grown at low nutrient level, and panels B and D were for plants grown at high nutrient level. Narrow bars depict means and SE for each population; two thicker bars in the center are means and SE for all populations from each range. * indicates significant difference between ranges at each nutrient level according to one-way nested ANCOVA (*P*<0.05; Tables S2, S4 in File S1).

*Chromolaena odorata* plants of the populations from nonnative ranges had lower root to shoot ratios than plants of the populations from native ranges grown in either monoculture or competition in both the low and the high nutrient treatments (Fig. 3; Table S4 in File S1). Addition of nutrients significantly decreased root to shoot ratios of plants from both ranges (Table S2 in File S1).

**Biogeographical Differences in Defensive Traits**

**Figure 4:**
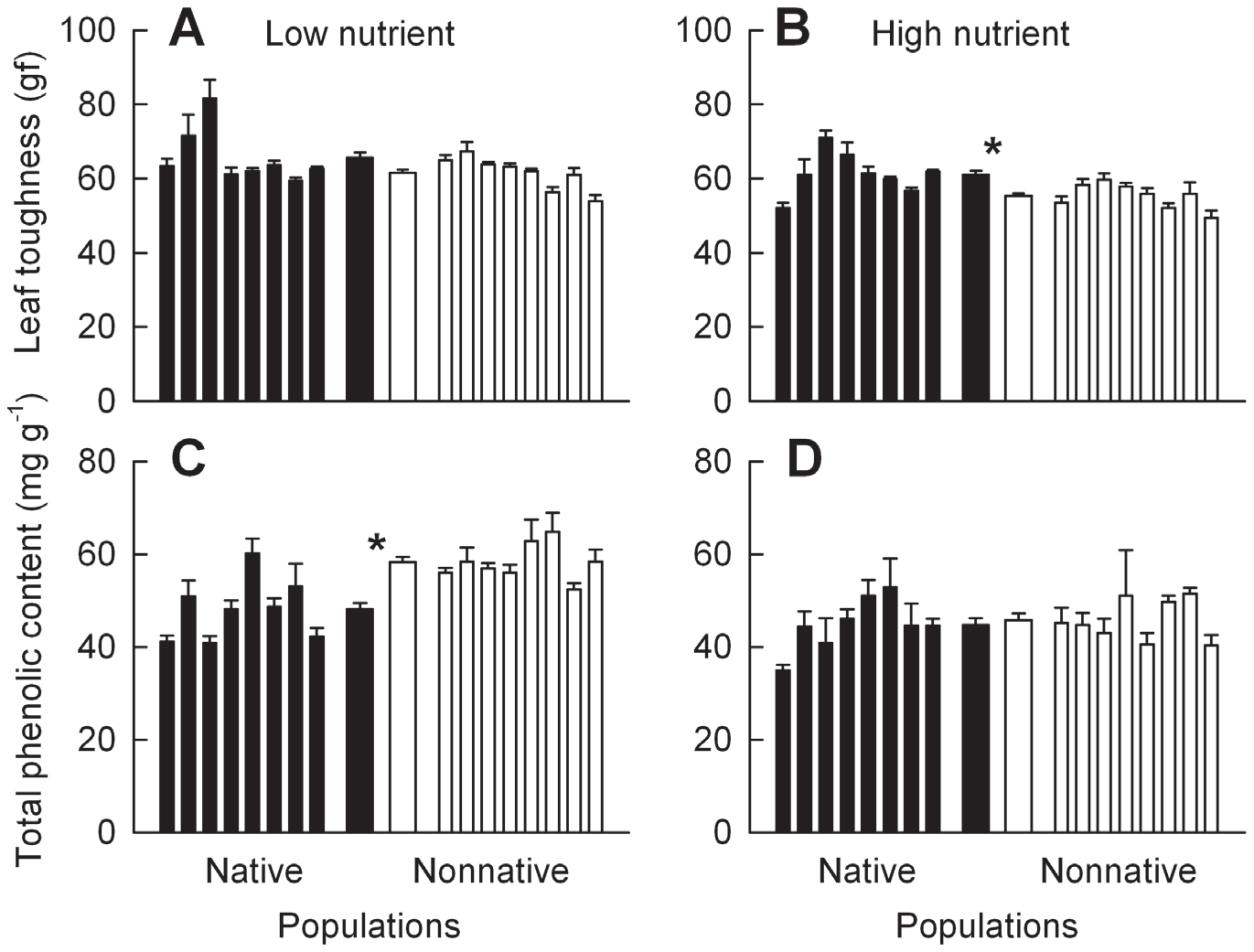
Differences in leaf toughness and phenolics between *Chromolaena odorata* plants of the populations from native versus nonnative ranges. Panels A and C were for plants grown at low nutrient level (monoculture), and panels B and D were for plants grown at high nutrient level (monoculture). Narrow bars depict means and SE for each population; two thicker bars in the center are means and SE for all populations from each range. * indicates significant difference between ranges at each nutrient level according to one-way nested ANOVA (*P*<0.05; Tables S3, S5 in File S1).

**Figure 5:**
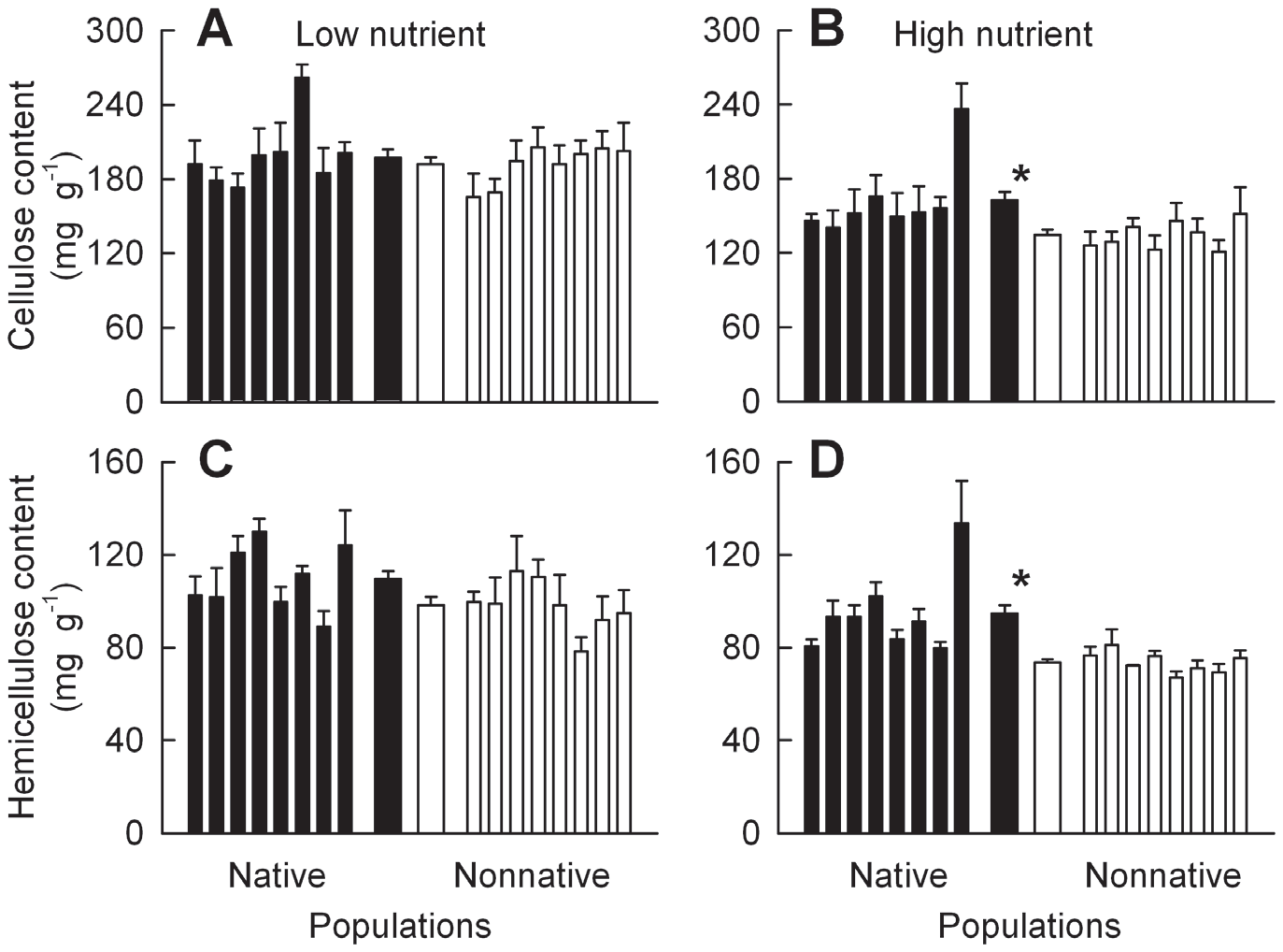
Differences in leaf cellulose and hemicellulose between *Chromolaena odorata* plants of the populations from native versus nonnative ranges. Panels A and C were for plants grown under low nutrient level (monoculture), and panels B and D were for plants grown under high nutrient level (monoculture). Narrow bars depict means and SE for each population; two thicker bars in the center are means and SE for all populations from each range. * indicates significant difference between ranges at each nutrient level according to one-way nested ANOVA (*P*<0.05; Tables S3, S5 in File S1).

In the low nutrient treatment, *C. odorata* plants of the populations from nonnative ranges had higher leaf phenolic content than plants of the populations from native ranges, while leaf toughness, leaf cellulose content, and hemicellulose content were not significantly different between ranges (Figs. 4A, 4C; 5A, C; Table S5 in File S1). Soil nutrient addition significantly decreased the defensive traits described above (Table S3 in File S1; Figs 4, 5). In the high nutrient treatment, *C. odorata* plants of the populations from nonnative ranges had lower leaf toughness, leaf cellulose content and hemicellulose content but similar leaf phenolic content compared with plants of the populations from native ranges (Figs. 4B, D; 5B, D).

## Discussion

Many studies have found that invasive plant species display increased vigor and/or decreased defense against natural enemies in their nonnative ranges relative to their native ranges [7], [9], [15], [41], [42]. These changes are consistent with the predictions of the EICA hypothesis, and are generally attributed to adaptive responses of invasive species to release from their natural enemies in nonnative ranges [2]. However, our results indicate that the biogeographical differences in competitive and defensive abilities between *C. odorata* plants from nonnative versus native ranges may also be associated with their responses to abiotic environments. In response to increased availability of soil nutrients in introduced ranges, *C. odorata* plants from the nonnative ranges had lower root to shoot ratios than plants from the native ranges, which may account for the inconsistent results found in different nutrient treatments for the biogeographical differences in competitive ability.

Like many other alien plant species, *C. odorata* generally invades disturbed habitats, where soil nutrient availability is relatively high and interspecific competition is relatively weak [31], [32]. In nonnative ranges, invasive plants including *C. odorata* often form dense monocultures with strong intraspecific competition [17], [29], [43]. Invasive plants may make adaptive changes in responses to these new environments. The lower root to shoot ratios of *C. odorata* plants of the populations from nonnative ranges compared with plants of the populations from native ranges may be an example of such changes. It is known that plants generally decrease root to shoot ratios with increasing availability of soil nutrients (especially nitrogen) [24], [33], [44], which is associated with the effects of miRNA on auxin response factors [45]. Genotypes with low root to shoot ratios may be selected for in environments with high levels of soil nutrients.

In habitats with high levels of soil nutrients, low root to shoot ratios may help plants to increase biomass accumulation by reducing root respiratory carbon loss without influencing soil resource uptake [46]. The low root to shoot ratio may also promote plant carbon accumulation by increasing the proportion of photosynthetic organs such as leaves and support organs [47], [48]. Thus, besides other characteristics such as allelopathy, biotic resistance, and acclimation ability [49], [50], the lower root to shoot ratio may also contribute to successful invasion of *C. odorata*. However, in habitats with limited availability of soil nutrients, belowground competition for soil resources becomes stronger and competition for aboveground resources such as light becomes weaker. Thus, the lower root to shoot ratios of *C. odorata* plants from nonnative ranges indicated that they were less able to capture soil resources than were plants from native ranges, providing a possible explanation for their lower competitive ability when grown in the low nutrient treatment. Consistently, competitive ability increased with increasing availability of soil nutrients in the smaller rooted *C. odorata* plants from nonnative ranges. In soils with greater resources, *C. odorata* plants from nonnative ranges had greater interspecific competitive ability than did plants from the native ranges [50]. These results partly support the body of evidence suggesting that disturbance and greater levels of soil nutrients provide at least some nonnative plants with a competitive advantage over resident native plant species [21].

In the high nutrient treatment, *C. odorata* plants from nonnative relative to native ranges decreased resource investment in quantitative chemical defense, as judged by the decreased leaf toughness and hemicellulose and cellulose contents. The results indicate that plants from the nonnative ranges have decreased defense against enemies. In support of this, *C. odorata* plants from nonnative ranges were reported to experience more damage from natural enemies than plants in portions of its native ranges [50]. According to the EICA hypothesis, the reduced expression of defense traits in *C. odorata* plants from nonnative ranges may result from adaptive responses to enemy release. The invader has grown for more than 150 years in nonnative ranges with few enemies [34]. However, we could not exclude the potential effects of the novel abiotic environments in nonnative ranges on the decreased investment in defense. Plants can also evolve decreased defenses in responses to increased soil nutrient availability [27], [36]. Decreased defense of *C. odorata* plants from nonnative ranges may be a result of enemy release interacting with increased availability of soil nutrients.

*Chromolaena odorata* plants from both ranges increased expression of defense traits when grown in the low availability of soil nutrients. This is consistent with the fact that replacement of resources lost due to herbivory is more costly in low nutrient environments than in higher nutrient environments [27]. However, *C. odorata* plants from nonnative ranges growing in the low nutrient treatment did not have lower expression of defense traits. In contrast, *C. odorata* plants of the populations from nonnative ranges had a higher leaf phenolic content. Phenolics are often considered to be quantitative chemicals (providing defense against specialists), decreasing leaf palatability and digestibility. However, some phenolic compounds are toxic and can act as qualitative chemicals to defend against generalists [36]. If this is the case for *C. odorata*, the decreased phenolic content of *C. odorata* plants from nonnative ranges is consistent with the modified EICA hypothesis, which predicts that invasive plants may evolve increased resistance against generalists after introduction [36].

Our common garden experiment excluded the confounding effects of phenotypic plasticity, but could not exclude the founder effects, as in almost all studies of the evolution of invasive species [42], [50]. Evidence for biogeographical differentiation in competitive abilities and defensive traits was found in *C. odorata* in the present study. However, we do not know with certainty whether this differentiation is related to adaptive evolution after introduction. We must compare nonnative populations with their specific source populations to answer this question. Unfortunately, the source populations of invasive species are rarely known.

Our results highlight the importance of conducting competition experiments in more than one environment when testing the EICA hypothesis. Growth in the absence of competition may not be a reliable predictor of competitive ability, especially when invasive plants have acclimated to habitats with relative low levels of interspecific competition. *Chromolaena odorata* plants from nonnative ranges were taller in competition-free environments but had similar or even lower competitive ability compared with plants from native ranges, which is consistent with results from other invasive plants [17], [18]. Our results indicate that performing experiments in more than one environment helps to reveal potential adaptive changes in invasive species and the mechanisms underlying these changes.

### Conclusions

In low nutrient conditions, *C. odorata* plants from nonnative ranges showed significantly lower competitive ability and higher total leaf phenolic content than did plants from native ranges, which is contrary to the EICA hypothesis. In high nutrient conditions, *C. odorata* plants from nonnative ranges showed similar competitive ability and lower leaf toughness and cellulosic contents compared with plants from native ranges, which is consistent with one of the predictions (decreased defense after introduction) of the EICA hypothesis. These inconsistent results may be associated with the adaptive shift in biomass allocation of plants from nonnative ranges. In the low nutrient treatment, the reduced root to shoot ratios of *C. odorata* plants from nonnative ranges contributed to their decreased competitive ability. Also, the decreased leaf toughness and cellulosic contents of these plants did not lead to a greater competitive ability for *C. odorata* plants from nonnative ranges in the treatment with added nutrients. Our results suggest that adaptive shift in biomass allocation influences biogeographical differences in competitive abilities of invasive plants, and provide a possible mechanism for the interaction between range and environment, which was found in the few studies that tested the EICA hypothesis in different environments.

## Supporting Information

File S1 Figure S1, Differences in plant height and total biomass between *Chromolaena odorata* plants from native versus nonnative ranges when grown with competition. Table S1, Background information on sample populations of *Chromolaena odorata*. Table S2, Effects of range, nutrient, and their interaction on variables related to growth according to two-way nested ANOVAs. Table S3, Effects of range, nutrient, and their interaction on variables related to resistance according to two-way nested ANOVAs. Table S4, Effects of range on variables related to growth measured in each nutrient treatment according to one-way nested ANOVAs. Table S5, Effects of range on variables related to resistance measured in each nutrient treatment according to one-way nested ANOVAs. Table S6, Effects of range, nutrient, and their interaction on variables related to growth according to two-way nested ANOVAs. Table S7, Effects of range on variables related to growth measured in each nutrient treatment according to one-way nested ANOVAs.
(DOC)
