# Supplementary material for: Differences in Competitive Ability between Plants from Nonnative and Native Populations of a Tropical Invader Relates to Adaptive Responses in Abiotic and Biotic Environments
Source: PLoS One. 2013 Aug 16;8(8):e71767. doi: 10.1371/journal.pone.0071767 (PMC3745391; doi:10.1371/journal.pone.0071767)
Supplement: File S1 — Figure S1, Differences in plant height and total biomass between Chromolaena odorata plants from native versus nonnative ranges when grown with competition. Table S1, Background information on sample populations of Chromolaena odorata. Table S2, Effects of range, nutrient, and their interaction on variables related to growth according to two-way nested ANOVAs. Table S3, Effects of range, nutrient, and their interaction on variables related to resistance according to two-way nested ANOVAs. Table S4, Effects of range on variables related to growth measured in each nutrient treatment according to one-way nested ANOVAs. Table S5, Effects of range on variables related to resistance measured in each nutrient treatment according to one-way nested ANOVAs. Table S6, Effects of range, nutrient, and their interaction on variables related to growth according to two-way nested ANOVAs. Table S7, Effects of range on variables related to growth measured in each nutrient treatment according to one-way nested ANOVAs. (DOC) [file pone.0071767.s001.doc]

**Figure S1. Differences in plant height and total biomass between *Chromolaena odorata* plants from native versus nonnative ranges when grown with competition.** Panels A and C were for plants grown at low nutrient level, and panels B and D were for plants grown at high nutrient level. Narrow bars depict means and SE for each population; two thicker bars in the center are means and SE for all populations from each range. * indicates significant difference between ranges at each nutrient level according to one-way nested ANOVA (*P* <0.05; Tables S6, S7).

**Table S1.** Information on 16 sample populations of *Chomolaena odorata.*

| Range | Location | Latitude | Longitude | Elevation (m) | Annual mean temperature (°C) | Annual precipitation (mm) |
| --- | --- | --- | --- | --- | --- | --- |
| Native |  |  |  |  |  |  |
|  | St Andrew, Jamaica | N 18°02′ | W 76°43′ | 747 | 22.3 | 2440 |
|  | Ponce, Puerto Rico | N 18°11′ | W 66°51′ | 300 | 21.9 | 2471 |
|  | Manati, Puerto Rico | N 18°12′ | W 66°06′ | 390 | 23.2 | 1929 |
|  | Pinar del Río, Cuba | N 22°45′ | W 82°50′ | 565 | 21.8 | 1792 |
|  | Miami, Florida, USA | N 25°38′ | W 80°20′ | 1~5 | 23.9 | 1407 |
|  | Collier, Florida, USA | N 25°52′ | W 80°29′ | 1~5 | 23.4 | 1324 |
|  | Broward, Florida, USA | N 26°08′ | W 80°06′ | 1~5 | 24.0 | 1508 |
|  | Martin, Florida, USA | N 27°06′ | W 80°15′ | 1~5 | 23.2 | 1382 |
| Invasive |  |  |  |  |  |  |
|  | Vientiane, Laos | N 17°58′ | E 102°37′ | 170 | 25.4 | 1622 |
|  | Shanya, Hainan, China | N 18°19′ | E 109°12′ | 23 | 25.6 | 1296 |
|  | ConCuong, Nghe An, Vietnam | N 19°03′ | E 104°53′ | 55 | 24.3 | 1528 |
|  | Menglun, Yunnan, China | N 21°56′ | E 101°15′ | 544 | 23.1 | 1363 |
|  | Puer, Yunnan, China | N 22°46′ | E 100°56′ | 1380 | 18.6 | 1520 |
|  | Nanning, Guangxi, China | N 22°51′ | E 108°09′ | 72 | 22.4 | 1422 |
|  | Baise, Guangxi, China | N 23°53′ | E 106°38′ | 140 | 23.4 | 1127 |
|  | Jingdong, Yunnan, China | N 24°17′ | E 100°50′ | 1263 | 20.5 | 958 |

Note: Annual mean temperature and annual precipitation were obtained from the WORLDCLIM data set with a resolution of 1 km^2^. For detailed please see: Hijmans RJ, Cameron SE, Parra JL, Jones PG, Jarvis A (2005) Very high resolution interpolated climate surfaces for global land areas. International Journal of Climatology 25: 1965-1978.

**Table S2.** Effects of range, nutrient, and their interaction on variables related to growth according to two-way nested ANOVAs.

| Variables | *Df* | type III SS | *MS* | *F*-value | *P*-value | *Df* | type III SS | *MS* | *F*-value | *P*-value |
| --- | --- | --- | --- | --- | --- | --- | --- | --- | --- | --- |
|  | **Plant height** | | | | | **Change in plant height with competition** | | | | |
| Range (R) | 1 | 2279.22 | 2279.22 | 3.24 | 0.094 | 1 | 2.84 | 2.84 | 11.24 | 0.005 |
| Nutrient (N) | 1 | 21193.32 | 21193.32 | 122.43 | <0.001 | 1 | 3.12 | 3.12 | 21.33 | <0.001 |
| R × N | 1 | 1509.79 | 1509.79 | 8.72 | 0.011 | 1 | 1.64 | 1.64 | 11.22 | 0.005 |
| Population (R) | 14 | 9854.60 | 703.90 | 3.87 | <0.001 | 14 | 3.54 | 0.25 | 10.05 | <0.001 |
| P (R) × N | 14 | 2423.41 | 173.10 | 0.95 | 0.507 | 14 | 2.05 | 0.15 | 5.81 | <0.001 |
| Error | 122 | 22201.66 | 181.98 |  |  | 1200 | 30.20 | 0.03 |  |  |
|  | **Total biomass** | | | | | **Change in total biomass with competition** | | | | |
| Range (R) | 1 | 494.28 | 494.28 | 1.65 | 0.220 | 1 | 6.57 | 6.57 | 14.38 | 0.002 |
| Nutrient (N) | 1 | 135092.11 | 135092.11 | 854.58 | <0.001 | 1 | 0.26 | 0.26 | 1.02 | 0.330 |
| R × N | 1 | 825.60 | 825.60 | 5.22 | 0.038 | 1 | 3.12 | 3.12 | 12.16 | 0.004 |
| Population (R) | 14 | 4202.78 | 300.20 | 2.11 | 0.015 | 14 | 6.40 | 0.46 | 9.52 | <0.001 |
| P (R) × N | 14 | 2213.13 | 158.08 | 1.11 | 0.354 | 14 | 3.60 | 0.26 | 5.36 | <0.001 |
| Error | 122 | 17334.01 | 142.08 |  |  | 1200 | 57.58 | 0.05 |  |  |
|  | **Root : shoot ratio in monoculture** | | | | | **Root : shoot ratio in competition** | | | | |
| Range (R) | 1 | 0.14 | 0.14 | 9.99 | 0.007 | 1 | 1.25 | 1.25 | 8.55 | 0.011 |
| Nutrient (N) | 1 | 0.10 | 0.10 | 21.68 | <0.001 | 1 | 3.05 | 3.05 | 123.87 | <0.001 |
| R × N | 1 | 0.00 | 0.00 | 0.37 | 0.551 | 1 | 0.01 | 0.01 | 0.50 | 0.491 |
| Population (R) | 14 | 0.20 | 0.01 | 3.68 | <0.001 | 14 | 2.05 | 0.15 | 12.04 | <0.001 |
| P (R) × N | 14 | 0.06 | 0.00 | 1.14 | 0.331 | 14 | 0.34 | 0.02 | 2.03 | 0.013 |
| Error | 122 | 0.47 | 0.00 |  |  | 1160 | 14.09 | 0.01 |  |  |

Note: Range, nutrient, and their interaction were treated as fixed factors; population nested within range [P (R)] and the interaction of P (R) by nutrient as random factors; initial plant height as a covariate when analyzing plant height, total biomass, change in plant height with competition, and change in total biomass with competition; and total biomass as a covariate when analyzing root : shoot ratio.

**Table S3.** Effects of range, nutrient, and their interaction on variables related to resistance according to two-way nested ANOVAs.

| Variables | *Df* | type III SS | *MS* | *F*-value | *P*-value | *Df* | type III SS | *MS* | *F*-value | *P*-value |
| --- | --- | --- | --- | --- | --- | --- | --- | --- | --- | --- |
|  | **Leaf toughness (gf)** | | | | | **Total phenolic content (mg g^-1^)** | | | | |
| Range (R) | 1 | 682.55 | 682.55 | 4.49 | 0.052 | 1 | 0.09 | 0.09 | 5.69 | 0.032 |
| Nutrient (N) | 1 | 772.62 | 772.62 | 21.34 | <0.001 | 1 | 0.20 | 0.20 | 33.30 | <0.001 |
| R × N | 1 | 110.13 | 110.13 | 3.04 | 0.103 | 1 | 0.05 | 0.05 | 9.18 | 0.009 |
| Population (R) | 14 | 2127.82 | 151.99 | 9.87 | <0.001 | 14 | 0.22 | 0.02 | 3.79 | <0.001 |
| P R) × N | 14 | 506.98 | 36.21 | 2.35 | 0.006 | 14 | 0.08 | 0.01 | 1.41 | 0.158 |
| Error | 122 | 1879.24 | 15.40 |  |  | 123 | 0.51 | 0.00 |  |  |
|  | **Leaf cellulose content (mg g^-1^)** | | | | | **Leaf hemicellulose content (mg g^-1^)** | | | | |
| Range (R) | 1 | 0.08 | 0.08 | 5.31 | 0.037 | 1 | 0.23 | 0.23 | 11.30 | 0.005 |
| Nutrient (N) | 1 | 0.60 | 0.60 | 52.67 | <0.001 | 1 | 0.31 | 0.31 | 61.83 | <0.001 |
| R × N | 1 | 0.04 | 0.04 | 3.27 | 0.092 | 1 | 0.02 | 0.02 | 4.56 | 0.051 |
| Population (R) | 14 | 0.20 | 0.01 | 1.86 | 0.037 | 14 | 0.28 | 0.02 | 3.15 | <0.001 |
| P (R) × N | 14 | 0.16 | 0.01 | 1.47 | 0.133 | 14 | 0.07 | 0.01 | 0.78 | 0.69 |
| Error | 124 | 0.97 | 0.01 |  |  | 123 | 0.79 | 0.01 |  |  |

Note: Range, nutrient, and their interaction were treated as fixed factors; population nested within range [P (R)] and the interaction of P (R) by nutrient as random factors.

**Table S4.** Effects of range on variables related to growth measured in each nutrient treatment according to one-way nested ANOVAs.

|  | **Low nutrient treatment** | | | | |  | **High nutrient treatment** | | | | |
| --- | --- | --- | --- | --- | --- | --- | --- | --- | --- | --- | --- |
| Variables | *Df* | type III SS | *MS* | *F*-value | *P*-value |  | *Df* | type III SS | *MS* | *F*-value | *P*-value |
| **Plant height** | | | | | | | | | | | |
| Range | 1 | 3402.90 | 3402.90 | 6.23 | 0.025 |  | 1 | 65.65 | 65.65 | 0.21 | 0.658 |
| Population (Range) | 14 | 7647.59 | 546.26 | 2.26 | 0.015 |  | 14 | 4479.87 | 319.99 | 2.53 | 0.006 |
| Error | 60 | 14473.34 | 241.22 |  |  |  | 61 | 7702.57 | 126.27 |  |  |
| **Total biomass (g)** | | | | | | | | | | | |
| Range | 1 | 75.19 | 75.19 | 2.83 | 0.114 |  | 1 | 1410.01 | 1410.01 | 3.13 | 0.098 |
| Population (Range) | 14 | 371.32 | 26.52 | 1.97 | 0.037 |  | 14 | 6312.71 | 450.91 | 1.71 | 0.077 |
| Error | 59 | 795.95 | 13.49 |  |  |  | 61 | 16095.10 | 263.85 |  |  |
| **Change in plant height with competition** | | | | | | | | | | | |
| Range | 1 | 6.28 | 6.28 | 16.21 | 0.001 |  | 1 | 0.06 | 0.06 | 0.42 | 0.526 |
| Population (Range) | 14 | 5.42 | 0.39 | 9.44 | <0.001 |  | 14 | 2.02 | 0.14 | 7.79 | <0.001 |
| Error | 612 | 25.11 | 0.04 |  |  |  | 588 | 10.90 | 0.02 |  |  |
| **Change in total biomass with competition** | | | | | | | | | | | |
| Range | 1 | 7.17 | 7.17 | 18.33 | <0.001 |  | 1 | 0.28 | 0.28 | 1.30 | 0.274 |
| Population (Range) | 14 | 5.48 | 0.39 | 10.02 | <0.001 |  | 14 | 3.02 | 0.22 | 4.93 | <0.001 |
| Error | 612 | 23.89 | 0.04 |  |  |  | 588 | 25.75 | 0.04 |  |  |
| **Root to shoot mass ratio in monoculture** | | | | | | | | | | | |
| Range | 1 | 0.05 | 0.05 | 8.55 | 0.011 |  | 1 | 0.04 | 0.04 | 9.43 | 0.008 |
| Population (Range) | 14 | 0.08 | 0.08 | 1.78 | 0.063 |  | 14 | 0.06 | 0.00 | 2.67 | 0.004 |
| Error | 60 | 0.18 | 0.00 |  |  |  | 61 | 0.10 | 0.00 |  |  |
| **Root : shoot ratio in competition** | | | | | | | | | | | |
| Range | 1 | 0.55 | 0.55 | 11.75 | 0.004 |  | 1 | 0.54 | 0.54 | 4.84 | 0.045 |
| Population (Range) | 14 | 0.65 | 0.05 | 3.59 | <0.001 |  | 14 | 1.55 | 0.11 | 10.22 | <0.001 |
| Error | 608 | 7.85 | 0.01 |  |  |  | 550 | 5.98 | 0.01 |  |  |

Note: Range was treated as a fixed factor; population nested within range as a random factor; initial height as a covariate when analyzing plant height, total biomass, change in plant height with competition, and change in total biomass in competition; total biomass as a covariate when analyzing root : shoot ratio.

**Table S5.** Effects of range on variables related to resistance measured in each nutrient treatment according to one-way nested ANOVAs.

|  | **Low nutrient treatment** | | | | |  | **High nutrient treatment** | | | | |
| --- | --- | --- | --- | --- | --- | --- | --- | --- | --- | --- | --- |
| Variables | *Df* | type III SS | *MS* | *F*-value | *P*-value |  | *Df* | type III SS | *MS* | *F*-value | *P*-value |
| **Leaf toughness (gf)** | | | | | | | | | | | |
| Range | 1 | 0.01 | 0.01 | 1.68 | 0.216 |  | 1 | 0.03 | 0.03 | 6.03 | 0.028 |
| Population (Range) | 14 | 0.10 | 0.01 | 7.21 | <0.001 |  | 14 | 0.08 | 0.01 | 5.81 | <0.001 |
| Error | 64 | 0.07 | 0.00 |  |  |  | 61 | 0.06 | 0.00 |  |  |
| **Total phenolic content (mg g^-1^)** | | | | | | | | | | | |
| Range | 1 | 1967.45 | 1967.45 | 13.00 | 0.003 |  | 1 | 2.65 | 2.65 | 0.02 | 0.882 |
| Population (Range) | 14 | 2118.76 | 151.34 | 3.84 | <0.001 |  | 14 | 1634.97 | 116.78 | 2.50 | 0.007 |
| Error | 62 | 2424.77 | 39.11 |  |  |  | 60 | 2798.07 | 46.63 |  |  |
| **Leaf cellulose content (mg g^-1^)** | | | | | | | | | | | |
| Range | 1 | 0.00 | 0.00 | 0.33 | 0.574 |  | 1 | 0.11 | 0.11 | 6.98 | 0.019 |
| Population (Range) | 14 | 0.15 | 0.01 | 1.56 | 0.118 |  | 14 | 0.22 | 0.02 | 1.82 | 0.056 |
| Error | 62 | 0.43 | 0.01 |  |  |  | 62 | 0.54 | 0.01 |  |  |
| **Leaf hemicellulose content (mg g^-1^)** | | | | | | | | | | | |
| Range | 1 | 2660.4 | 2660.4 | 3.45 | 0.084 |  | 1 | 0.20 | 0.20 | 15.47 | 0.002 |
| Population (Range) | 14 | 10785.05 | 770.36 | 1.73 | 0.072 |  | 14 | 0.18 | 0.01 | 3.26 | 0.001 |
| Error | 62 | 27610.53 | 445.33 |  |  |  | 61 | 0.24 | 0.00 |  |  |

Note: Range was treated as a fixed factor; population nested within range as a random factor.

**Table S6.** Effects of range, nutrient, and their interaction on variables related to growth according to two-way nested ANOVAs.

| Variables | *Df* | type III SS | *MS* | *F*-value | *P*-value | *Df* | type III SS | *MS* | *F*-value | *P*-value |
| --- | --- | --- | --- | --- | --- | --- | --- | --- | --- | --- |
|  | **Plant height** | | | | | **Total biomass** | | | | |
| Range (R) | 1 | 5785.97 | 5785.97 | 3.94 | 0.067 | 1 | 8110.31 | 8110.31 | 7.54 | 0.016 |
| Nutrient (N) | 1 | 255728.71 | 255728.71 | 631.99 | <0.001 | 1 | 227444.88 | 227444.88 | 776.19 | <0.001 |
| R × N | 1 | 992.16 | 992.16 | 2.45 | 0.140 | 1 | 1219.93 | 1219.93 | 4.16 | 0.0061 |
| Population (R) | 14 | 20554.41 | 1468.17 | 6.28 | <0.001 | 14 | 15064.21 | 1076.02 | 14.26 | <0.001 |
| P (R) × N | 14 | 5664.93 | 404.64 | 1.73 | 0.044 | 14 | 4102.41 | 293.03 | 3.88 | <0.001 |
| Error | 1160 | 271011.32 | 233.63 |  |  | 1160 | 87519.51 | 75.45 |  |  |

Note: Range, nutrient, and their interaction were treated as fixed factors; population nested within range [P (R)] and the interaction of P (R) by nutrient as random factors; initial plant height as a covariate when analyzing plant height, total biomass.

**Table S7.** Effects of range on variables related to growth measured in each nutrient treatment according to one-way nested ANOVAs.

|  | **Low nutrient treatment** | | | | |  | **High nutrient treatment** | | | | |
| --- | --- | --- | --- | --- | --- | --- | --- | --- | --- | --- | --- |
| Variables | *Df* | type III SS | *MS* | *F*-value | *P*-value |  | *Df* | type III SS | *MS* | *F*-value | *P*-value |
| **Plant height** | | | | | | | | | | | |
| Range | 1 | 7484.00 | 7484.00 | 6.39 | 0.024 |  | 1 | 586.67 | 586.67 | 0.68 | 0.423 |
| Population (Range) | 14 | 16392.09 | 1170.86 | 4.51 | <0.001 |  | 14 | 12069.16 | 862.08 | 4.39 | <0.001 |
| Error | 607 | 157448.94 | 259.39 |  |  |  | 550 | 107945.15 | 196.26 |  |  |
| **Total biomass (g)** | | | | | | | | | | | |
| Range | 1 | 3.56 | 3.56 | 6.62 | 0.022 |  | 1 | 7711.26 | 7711.26 | 7.18 | 0.018 |
| Population (Range) | 14 | 7.52 | 0.54 | 16.50 | <0.001 |  | 14 | 15026.45 | 1073.32 | 7.56 | <0.001 |
| Error | 608 | 19.78 | 0.03 |  |  |  | 550 | 78136.47 | 142.07 |  |  |

Note: Range was treated as a fixed factor; population nested within range as a random factor; initial height as a covariate when analyzing plant height, total biomass.
